# Prevalence and risk factors accounting for true silent myocardial ischemia: a pilot case-control study comparing type 2 diabetic with non-diabetic control subjects

**DOI:** 10.1186/1475-2840-10-9

**Published:** 2011-01-21

**Authors:** Cristina Hernández, Jaume Candell-Riera, Andreea Ciudin, Gemma Francisco, Santiago Aguadé-Bruix, Rafael Simó

**Affiliations:** 1 CIBER de Diabetes y Enfermedades Metabólicas Asociadas (CIBERDEM; 2 RECAVA (Red de Enfermedades Cardiovasculares), Instituto de Salud Carlos III. Spain; 3 Diabetes Research Unit. Institut de Recerca Hospital Universitari Vall d'Hebron. Pg. Vall d'Hebron 119-129. 08035 Barcelona. Spain; 4 Cardiology Department. Hospital Universitari Vall d'Hebron. Pg. Vall d'Hebron 119-129. 08035 Barcelona. Spain; 5 Nuclear Medicine Department, Hospital Universitari Vall d'Hebron. Pg. Vall d'Hebron 119-129. 08035 Barcelona. Spain; 6 Universitat Autònoma de Barcelona, Pg. Vall d'Hebron 119-129, 08035 Barcelona. Spain

## Abstract

**Background:**

Given the elevated risk of cardiovascular events and the higher prevalence of silent coronary artery disease (CAD) in diabetic versus non-diabetic patients, the need to screen asymptomatic diabetic patients for CAD assumes increasing importante. The aims of the study were to assess prospectively the prevalence and risk factor predictors of true silent myocardial ischemia (myocardial perfusion defects in the absence of both angina and ST-segment depression) in asymptomatic type 2 diabetic patients.

**Methods:**

Stress myocardial perfusion gated SPECT (Single Photon Emission Computed Tomography) was carried out in 41 type 2 diabetic patients without history of cardiovascular disease (CVD) and 41 nondiabetic patients matched by age and gender.

**Results:**

There were no significant differences between the two groups regarding either the classic CVD risk factors or left ventricular function. True silent ischemia was detected in 21.9% of diabetic patients but only in 2.4% of controls (p < 0.01). The presence of myocardial perfusion defects was independently associated with male gender and the presence of diabetic retinopathy (DR). The probability of having myocardial perfusion defects in an asymptomatic diabetic patient with DR in comparison with diabetic patients without DR was 11.7 [IC95%: 3.7-37].

**Conclusions:**

True silent myocardial ischemia is a high prevalent condition in asymptomatic type 2 diabetic patients. Male gender and the presence of DR are the risk factors related to its development.

## Background

Patients with diabetes, in particular with type 2 diabetes, are at a 2- to 4- fold higher risk of cardiovascular mortality compared with nondiabetic subjects. In addition, diabetic patients are less likely to survive a first myocardial infarction than their nondiabetic peers. Therefore, early identification of coronary artery disease (CAD) in the diabetic population is needed. However, the fact that CAD is often asymptomatic in diabetic patients makes such identification a challenge. A number of studies have shown that silent myocardial ischemia - as evidenced by non-invasive tests such as the electrocardiogram stress test, myocardial scintigraphy or stress echocardiography- affects 20-50% of diabetic patients with additional risk factors [1]. The term of silent ischemia includes an entity named true silent myocardial ischemia or clandestine ischemia, which is characterized by myocardial perfusion defects in the absence of both angina and ST-segment depression > 1 mm during the exercise test. To the best of our knowledge there have been no studies addressed to determining its prevalence and the risk factors associated with its development.

In the largest study performed until now to evaluate the prevalence of silent myocardial ischemia in diabetic patients, the DIAD (Detection of Ischaemia in Assymptomatic Diabetics) study [2] patients with silent myocardial ischemia and patients with true silent myocardial ischemia were analyzed all together. After careful examination of the results one can deduce that 14% of the patients allocated to be screened with stress myocardial perfusion imaging had true silent myocardial ischemia. However, a control group of non-diabetic subjects was not included and, therefore, it is not possible to know how often assymptomatic diabetic patients have defects of myocardial perfusion in comparison with non-diabetic population.

The present case-control study was designed to assess prospectively the prevalence and risk factor predictors of true silent myocardial ischemia in asymptomatic type 2 diabetic patients without a history of cardiovascular disease (CVD).

## Methods

### Subjects

This study has been performed following the "Strengthening the Reporting of Observational Studies in Epidemiology" (STROBE) guidelines for reporting case-control studies [3].

#### Sample size calculation

We used the following formula for the sample size calculation:

(1) $$n=\frac{{\left[Z_{\alpha }\sqrt{2p(1-p)}+Z_{\beta }\sqrt{p_{1}(1-p_{1})+p_{2}(1-p_{2})}\right]}^{2}}{{\left(p_{1}-p_{2}\right)}^{2}}$$

n= estimated necessary sample size

Zα = Z-coefficient for Type I error

Zβ = Z-coefficient for Type II error

p_1 _= estimated proportion of myocardial perfussion defects in controls (non diabetic population)

p_2 _= estimated proportion of myocardial perfussion defects in cases (diabetic patients)

p = mean value of p_1 _and p_2_

For this purpose we estimated *a priori *that the prevalence of true silent ischemia would be of 2% (p_1 _= 0.02) in nondiabetic subjects and 20% (p_2 _= 0.20) in diabetic patients. We used a 5% of significance level and a power of 80%. The specific figures are displayed below:

(2) $$n=\frac{{\left[1.64\sqrt{0.22\left(0.89\right)}+0.84\sqrt{0.02\left(0.98\right)+0.20\left(0.80\right)}\right]}^{2}}{{\left(0.02-0.20\right)}^{2}}=36$$

Therefore, the minimum sample size was 36 subjects in each group.

#### Exclusion criteria

**Figure 1:**
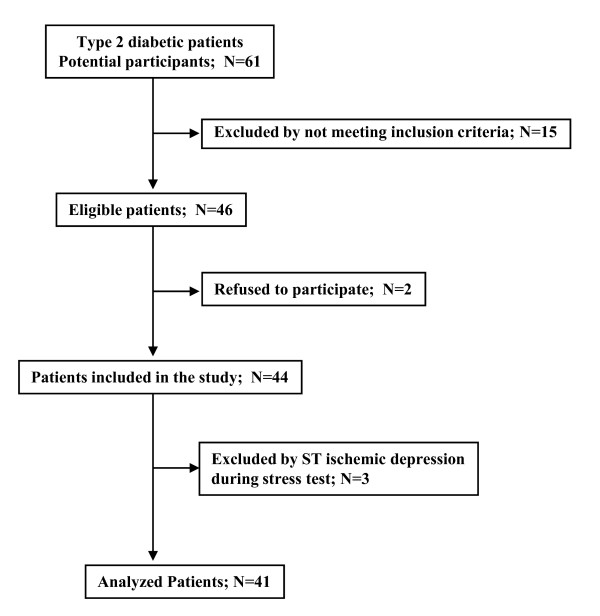
**Flow chart showing the patients included in the study**.

Exclusion criteria were: 1) history of CVD; 2) electrocardiographic evidence of Q-wave myocardial infarction, ischemic ST depression, T-wave changes, or complete left bundle branch block; 3) flat or downsloping ST segment depression > 1 mm at 80 ms after the J-point during an exercise test on a bicycle ergometer. On this basis 44 consecutive asymptomatic type 2 diabetic patients were propectively recruited from our outpatient diabetes clinic (Figure 1). Three patients were excluded from the study because they presented ST-segment depression > 1 mm during exercise test. Forty-one nondiabetic subjects matched by age and gender were selected as a control group.

This study was approved by the hospital's human ethics committee and conducted according to the principles expressed in the Declaration of Helsinki. Written informed consent was obtained from all patients.

#### Stress myocardial perfusion imaging

All patients were systematically screened with an exercise test on a bicycle ergometer and stress myocardial perfusion imaging using technetium-99m-methoxy-isobutyl-isonitrile single-photon emission computed tomography (SPECT). All patients did symptom-limited exercise on a stationary bicycle. One-day protocol gated SPECT was used: a first dose of 8 mCi, given 30-60 seconds before finishing exercise, and a second dose of 24 mCi, administered at rest, were separated by an interval of more than 45 minutes. The equipment used was a Siemens E-CAM dual head 90° gamma camera with a low energy high-resolution collimator and 180° semicircular orbit set in "step-and-shoot" mode, initiated at 45° right anterior oblique, with images every 3 degrees (25 second time frame). Acquisition was synchronized with the electrocardiogram R wave, with an 8 frame/cardiac cycle. Frames were reconstructed with this gamma camera using filtered backprojection (Butterworth filter, (order 10, cutoff frequency 0.35 for stress, and order 10, cutoff frequency 0,45 for rest). To quantify perfusion the left ventricle was divided into 17 segments, each of which was assigned a score from 0 to 4 (0 = normal perfusion, 1 = mild hypoperfusion, 2 = moderate hypoperfusion, 3 = severe hypoperfusion, and 4 = no uptake). The summed stress score and summed rest score were obtained, with the summed difference score being the difference between the two. An ischemic patient was defined as showing the presence of a summed difference score ≥ 2. A moderate perfusion defect was defined as a segmental score ≥ 2 in > 1 segment, and severe perfusion defect was defined as a segmental score ≥ 3 in > 1 segment in stress images [4,5]. Calculation of left ventricular ejection fraction and ventricular volumes was automatically performed during rest gated SPECT. Endocardial and epicardial boundaries were automatically traced using the quantitative QGS^® ^software (Cedars-Sinai Medical Center, Los Angeles, CA USA) [6].

#### Cardiovascular risk factors and diabetic retinopathy (DR) assessment

The cardiovascular risk factors evaluated were: obesity (defined as body mass index ≥30 Kg/m^2^), smoking habit (past or current), hypertension (defined as blood pressure > 140/90 mmHg or anti-hypertensive drug use), dyslipemia (defined as lipid abnormalities or lipid-lowering treatment), and diagnosis of CAD in parents or sibling before age 50.

Fundoscopic examination in mydriasis using slit lamp biomicroscopy was conducted by the same ophthalmologist who was unaware of the clinical status of the patient. In addition seven-standard field color retinal photographs were taken using a digital camera (Topcon TCR-50DX). The elapsed time between fundoscopic examination and gated SPECT was less than four weeks in all cases. For DR classification the International Clinical Diabetic Retinopathy Severity Scale was used [7]. Blood and urine samples were obtained for laboratory testing. The main clinical data of both groups are displayed in Table 1.

**Table 1:** Clinical, ergometric and scintigraphic characteristics of subjects included in the study

	Diabetic Patients	Controls	
	n = 41	n = 41	p
Age (years)	63.0 ± 5.4	61.1 ± 6.1	0.1
Male/Female (n)	11/30	11/30	0.59
Obesity^a ^(%)	48.8%	34.14%	0.26
Smoking^b ^(%)	19.5%	21.9%	0.95
Hypertension^c ^(%)	56%	36.6%	0.12
Dyslipemia^d ^(%)	73.1%	63.4%	0.33
Family history of CAD^e ^(%)	14.6%	17.1%	0.5
Duration of diabetes (years)	11.0 ± 7.2	-	
Retinopathy (%)	17.1%	-	
Nephropathy (%)	9.7%	-	
Neuropathy (%)	2.4%	-	
**Laboratory parameters**:			
Glucose (mg/dl)	162 ± 6	98 ± 9	<0.001
HbA1c (%)	8.2 ±1.3	5.8 ± 0.1	<0.001
AER (μg/minute)	6 [2-44]	5 [2-26]	0.73
Total-C (mg/dl)	206 ± 45	228 ± 39	0.06
HDL-C (mg/dl)	56 ± 10	55 ± 10	0.71
LDL-C (mg/dl)	121 ± 38	167 ± 19	<0.001
Triglycerides (mg/dl)	139 [65-242]	125 [66-175]	0.24
**Exercise test**:			
METs	6.74 ± 2.01	7.01 ± 1.78	0.60
Peak heart rate (bpm)	136 ± 14	138 ± 14	0.54
Peak systolic blood pressure (mmHg)	179 ± 24	172 ± 17	0.25
**Gated-SPECT**:			
Ejection fraction (%)	74.3 ± 11.1	72.6 ± 8.1	0.51
LV end-diastolic volume (ml)	57.6 ± 17.9	64.6 ± 14.6	0.12
LV end-systolic volume (ml)	16.4 ± 11.8	19.4 ± 8.6	0.30

Data are expressed as the mean ± SD or median [range]; bpm: beats per minute; LV: left ventricular;.METs: Estimated Metabolic Equivalent.

#### Statistical analysis

Bivariate associations were tested using *t *test and Fisher's exact test. To identify the factors independently related with clandestine ischemia a backward stepwise logistic regression analysis was performed.

## Results

Apart from diabetes there were no significant differences between the two groups regarding either the classic CVD risk factors (age, gender, smoking habit, dyslipemia, hypertension, and family history of CAD) or left ventricular function (Table 1). Diabetic patients presented lower levels of LDL-cholesterol than control subjects. The use of statins was higher in diabetic patients than in the control group (73% vs. 51%; p < 0.05).

**Figure 2:**
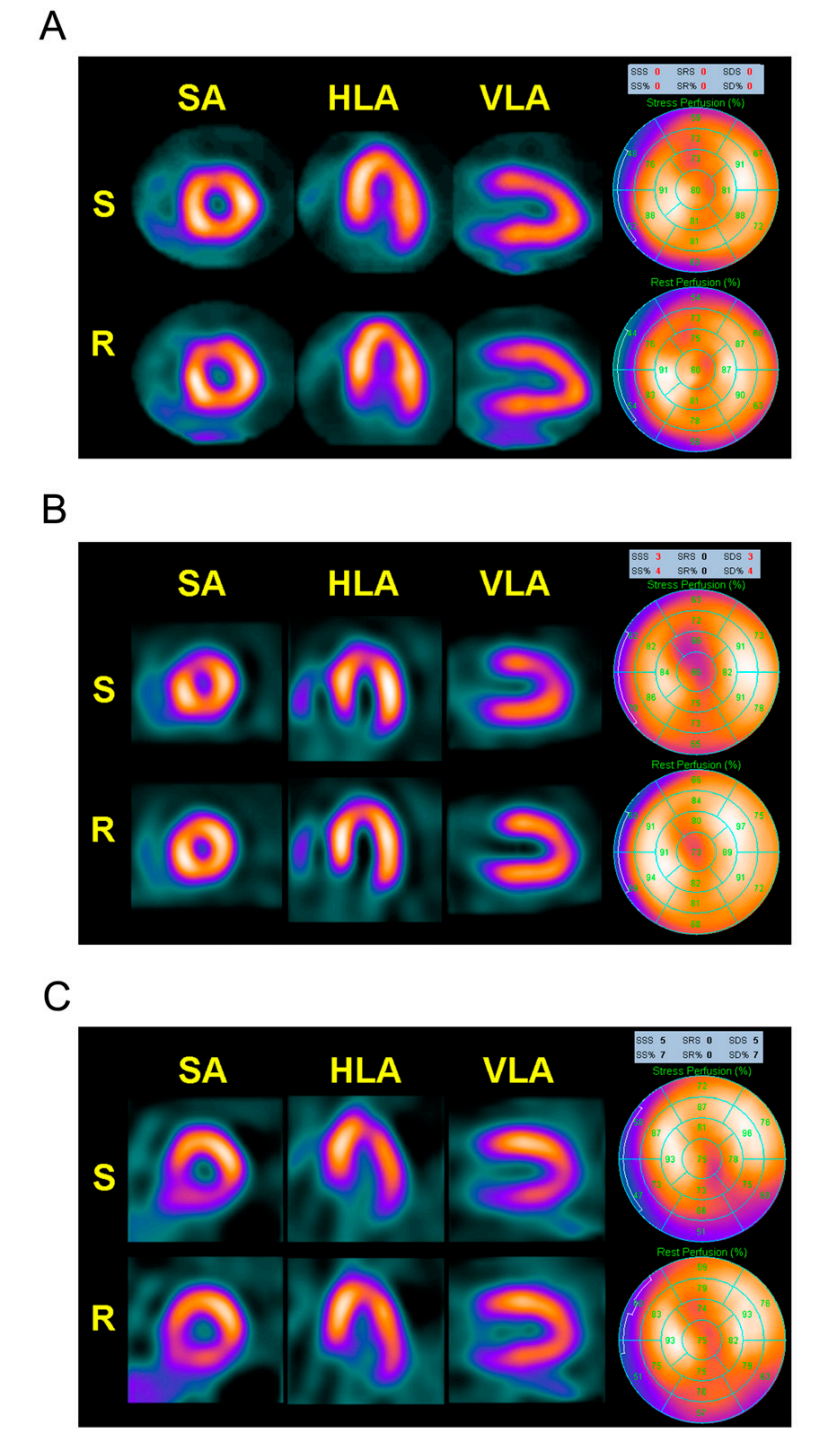
**Images corresponding to myocardial perfusion SPECT obtained in diabetic and non-diabetic control subjects.** A) Normal SPECT in a representative non-diabetic subject. B) Mild reversible defect in the left ventricular antero-apical region (SDS=3) in a representative type 2 diabetic patient. C) Moderate reversible defect in the left ventricular inferior region (SDS=5) in a representative type 2 diabetic patient. HLA: Horizontal Long Axis, R: Rest, S: Stress, SA: Short Axis, SDS: Summed Difference Score, SRS: Summed Rest Score, SSS: Summed Stress Score, VLA: Vertical Long Axis.

Three out of 44 recruited patients were excluded from the study because they presented ST-segment depression > 1 mm during exercise test (Figure 1). True silent myocardial ischemia was detected in 9 out of 41 (21.9%) type 2 diabetic patients (1 with moderate and 8 with mild perfusion defects) but only in one (mild perfusion defects) out of 41 controls (2.4%), (p < 0.01). Representative images of normal myocardial perfusion SPECT in non-diabetic subject, and mild and moderate perfusion defects detected in diabetic patients can be observed in figure 2. It should be noted that in the diabetic patient with inferior moderate perfusion defect a coronary angiography was performed and a <50% stenosis of right coronary artery was found.

In diabetic patients, the frequency of myocardial perfusion defects was 54.5% in male patients and 13.3% in females (p = 0.01). In addition, the prevalence of true silent ischemia in diabetic patients with DR was significantly higher than in diabetic patients without DR (50% vs. 13%; p = 0.04). On the other hand, DR was higher in patients with true silent myocardial ischemia than in patients without it (40% vs. 9.6%; p = 0.04).

In the logistic regression analysis, both male gender and DR were independently associated with the presence of true silent myocardial ischemia (Table 2).

**Table 2:** Logistic regression analysis showing the independent predictors of true silent myocardial ischemia in diabetic subjects

Variable	*Odds *ratio [95% CI]	*p*
Male gender (no/yes)	3.3 [1.2-8.5]	0.02
Diabetic retinopathy (no/yes)	11.7 [3.7-37]	0.03
Age (years)	1.03 [0.8-1.28]	0.79
HbA1C (%)	0.67 [-0.42-1.76]	0.57
Diabetes duration (years)	0.82 [0.59-1.05]	0.08
Plama cholesterol (mg/dl)	1.01 [0.98-1.04]	0.44
Smoking habit (no/yes)	0.20 [-3.01-3.41]	0.79

Apart from the variables associated with true silent ischemia in univariate analysis (gender, diabetic retinopathy), HbA1c, diabetes duration, and classic cardiovascular risk factors (age, plasma cholesterol, hypertension and smoking habit) were also entered in the logistic regression analysis.

Finally, we did not find any differences either in left ventricular volumes (end-systolic and end-diastolic) or in left ejection fraction between diabetic patients with and without myocardial perfusion defects measured during rest gated SPECT.

## Discussion

In this case-control study we evaluated for the first time the prevalence of true silent myocardial ischemia in asymptomatic type 2 diabetic patients in comparison with a non-diabetic control group. Risk factors of CVD were similar between both groups. However, diabetic patients presented lower levels of LDL-cholesterol, this being accounted by the use of statins. We found that the prevalence of true silent myocardial ischemia is strikingly higher than in their nondiabetic peers. As previously reported in silent myocardial ischemia [2], men are more likely to have perfusion defects. Moreover, as occurs in silent ischemia the number of traditional risk factors is not useful as a means of identifying patients with true silent myocardial ischemia [2,8,9].

One of the most important findings of the present study is that DR is independently associated with the presence of true silent myocardial ischemia. In this regard, DR has been recently recognized as an indicator of risk for CAD in diabetic patients [10-12] and it is predictive of cardiovascular mortality [13,14]. Our results are consistent with other observations supporting the concept that micro and macrovascular complications of diabetes share common pathogenic mecanisms beyond those related to the classical risk factors [10,13,15]. The common pathogenic mechanisms between micro and macrovascular complications are uncertain but there is emerging evidence to suggest that DR has common genetic linkages with systemic vascular complications [11]. In addition, endothelial dysfunction, platelet dysfunction, oxidative stress, inflammation, and advanced glycation end products are pathogenic factors for both micro and macrovascular complications in diabetic patients [13,15]. Furthermore, DR reflects widespread microcirculatory disease not only in the eye but also in vital organs elsewhere in the body such as myocardium. For all these reasons it is not surprising that type 2 diabetic patients with DR present a higher prevalence of myocardial perfusion defects in comparison with those patients without DR. However, in the DIAD study DR was not predictive of myocardial perfusion defects. The reasons why our findings are not coincident with DIAD remain to be elucidated, but it should be noted that in our study ophthalmoscopic examinations were performed in all cases within the same month as stress myocardial perfusion gated-SPECT and using the same method. By contrast, the methods for the assessment of DR described in the DIAD study were non-homogeneous and the time elapsed between fundoscopy and SPECT was not specified.

One might wonder which diabetic patients would benefit from a SPECT for ruling out a silent ischemia and whether the information drawn from this test might be beneficial in terms of CAD prognosis. This is important not only for the management of diabetic patients but also in economic terms. In this regard, the main aim of the DIAD study was to test the hypothesis that systematic screening with stress myocardial perfusion imaging would identify higher-risk individuals and beneficially affect their risk of myocardial infarction or cardiac death. The conclusion of the study was that the identification of patients with myocardial perfusion defects did not serve to lower their risk over 5 years of follow-up [16]. However, the DIAD study was not designed as a treatment trial and did not recommend coronary angiography or any specific treatment plan for patients with abnormal screening tests. In fact, the number of coronary angiograms performed during follow-up was similar in the screened and in the non-screened group. Moreover, the use of statins, angiotensin-converting enzyme inhibitors, antihypertensive and antihyperglycemic medications, and aspirin for primary medical prevention was the same for screened and non-screened patients. Therefore, it is logical that the screening process *per se *used in the DIAD study did not improve the clinical outcomes of diabetic patients. By contrast, in another randomised study also including asymptomatic type 2 diabetic patients, it has been observed that the rate of cardiac events was significantly lower in patients screened for silent myocardial ischemia and revascularized when necessary than in patients who were not screened [17]. In this study the protocol consisted in performing an exercice electrocardiogram test and dypiridamole stress echocardiography. If one of these tests were abnormal, a coronariography was then performed, and revascularization procedure was indicated in the case of stenoses ≥ 50% of vessel lumen. Using this scheduled management, no cardiac events occurred in patients who underwent revascularization procedures at the time of screening during the 5 years of follow-up. Finally, Mohagheghie et al recently found that 1/3 patients with diabetes mellitus without abnormal ECG findings and with myocardial perfusion defects presented occult CAD [18]

Although cost-effectiveness studies are still needed it is undeniable that type 2 diabetes could benefit from the identification of silent myocardial ischemia. In fact, a normal SPECT has a very high negative predictive value for myocardial infarction or cardiac death [19]. In addition, in the DIAD study asymptomatic diabetic patients with moderate or large myocardial perfusion defects had a 6-fold greater cardiac risk than those with normal studies or small defects [16]. However, the meaning of a positive SPECT in the setting of true silent myocardial ischemia, in particular when perfusion deficits are of mild intensity, remains to be elucidated. Meanwhile, in terms of clinical practice it would be reasonable to be especially rigorous in achieving a tight control of risk factors and blood glucose in these patients. In this regard it should be underlined that in the DIAD study, a more intensive treatment of cardiovascular risk factors was associated with the resolution of perfusion deficits [20].

The repercussion of true silent ischemia (in particular mild perfusion defects) on cardiac function in diabetic patients is a topic that remains to be elucidated. We did not find any differences either in left ventricular volumes (end-systolic and end-diastolic) or in left ejection fraction between diabetic patients with and without myocardial perfusion defects. However, it should be noted that diabetes can be associated with impaired myocardial performance assessed by echocardiographic tissue Doppler imaging even in the absence of significant CAD [21]. In addition, Hallén et al [22] have recently found in a relatively young and healthy type 2 diabetic population elevations of cardiac troponin with a clear trend toward adverse outcomes. Specific studies addressed to exploring whether these findings are more frequent in diabetic patients with myocardial perfusion defects are needed.

Given that a generalized screening of asymptomatic type 2 diabetic patients with stress myocardial perfusion imaging is not a realistic approach [23], we wanted to identify the most relevant risk factors associated with clandestine ischemia. We found that the presence of DR and male gender were independently related to the presence of myocardial perfusion defects in type 2 diabetic patients. The probability of having myocardial perfusion defects in an asymptomatic diabetic patient with DR was 11.7 [IC95%: 3.7-37] in comparison with diabetic patients without DR. Therefore, patients with DR are good target population for indicating SPECT. This information is important not only for the management of diabetic patients but also in terms of the economic burden. The current guidelines already identify the need for routine screening for DR. In addition to appropriate vision care, the detection of DR might now also warrant a fuller cardiac evaluation and closer follow-up to prevent the development of CAD.

Our results could also have therapeutic implications. There is now emerging evidence that intravitreal anti-VEGF (vascular endothelial growth factor) agents (ie. pegaptamib, ranibizumab, bevacizumab) are useful in the management of advanced DR [24]. However, anti-VEGF drugs injected into the vitreous could pass into systemic circulation and could compromise the capacity of collateral vessel development and cardiac remodelling [24,25]. It is well-established that high levels of VEGF and its receptors are observed in DR and in diabetic nephropathy, and this is particularly crucial for the development of DR. By contrast, low levels of VEGF and its receptors are found in the myocardium of diabetic patients [26,27], resulting in inadequate collateral formation. Therefore, diabetic patients with perfusion defects might be more prone to the development of myocardial ischemia if circulating VEGF is blocked. In consequence, patients with advanced DR requiring anti-VEGF treatment could be a selected group for whom stress myocardial perfusion gated SPECT could be specially indicated.

## Conclusions

We conclude that true silent myocardial ischemia is frequent in asymptomatic type 2 diabetic patients. In addition, DR is a high risk condition for true silent myocardial ischemia in asymptomatic type 2 diabetic patients, and point to these patients as a target to be screened for CAD. Further prospective studies evaluating the prognostic value of these findings and their impact in economical terms are warranted.

## Abbreviations

bpm: beats per minute; CAD: coronary artery disease; CVD: cardiovascular disease; DIAD: Detection of Ischaemia in Assymptomatic Diabetics; LV: left ventricular; METs: Estimated Metabolic Equivalent; DR: diabetic retinopathy; SPECT: Single Photon Emission Computed Tomography; VEGF: vascular endothelial growth factor.

## Competing interests

The authors declare that they have no competing interests.

## Authors' contributions

CH, JC and RS: conceived the study; CH, JC, AC, GF and SA: collecting and analyzing data; CH: statistical analysis and writing the draft; JC and RS: obtaining funding and revising the manuscript. All authors read and approved the final manuscript.
